# Role of the phosphocreatine system on energetic homeostasis in skeletal and cardiac muscles

**DOI:** 10.1590/S1679-45082014RB2741

**Published:** 2014

**Authors:** Lucas Guimarães-Ferreira

**Affiliations:** 1 Universidade Federal do Espírito Santo, Vitoria, ES, Brazil

**Keywords:** Phosphocreatine, Homeostasis, Muscle cells, Cardiac myocytes

## Abstract

Adenosine triphosphate is the present energy currency in the body, and is used in various cellular and indispensable processes for the maintenance of cell homeostasis. The regeneration mechanisms of adenosine triphosphate, from the product of its hydrolysis – adenosine diphosphate – are therefore necessary. Phosphocreatine is known as its quickest form of regeneration, by means of the enzyme creatine kinase. Thus, the primary function of this system is to act as a temporal energy buffer. Nevertheless, over the years, several other functions were attributed to phosphocreatine. This occurs as various isoforms of creatine kinase isoforms have been identified with a distinct subcellular location and functionally coupled with the sites that generate and use energy, in the mitochondria and cytosol, respectively. The present study discussed the central and complex role that the phosphocreatine system performs in energy homeostasis in muscle cells, as well as its alterations in pathological conditions.

## RESUMO

A adenosina trifosfato é a moeda corrente de energia no organismo, sendo utilizada em diversos processos celulares e indispensável para a manutenção da homeostase celular. Mecanismos de regeneração da adenosina trifosfato, a partir de seu produto de hidrólise – a adenosina difosfato – são, dessa forma, necessários. A fosfocreatina é conhecidamente sua fonte mais rápida de regeneração, por meio da enzima creatina quinase. Assim, a principal função desse sistema é atuar como um tampão temporal de energia. Entretanto, ao longo dos anos, diversas outras funções foram atribuídas à fosfocreatina. Isso ocorreu à medida que foram identificadas diversas isoformas da creatina quinase com localização subcelular distinta e acopladas de forma funcional aos sítios geradores e utilizadores de energia, na mitocôndria e citosol, respectivamente. O presente trabalho discutiu o papel central e complexo que o sistema da fosfocreatina desempenha na homeostase energética nas células musculares, bem como suas alterações em quadros patológicos.

## INTRODUCTION

Adenosine triphosphate (ATP) is the primary source of chemical energy, and its hydrolysis is highly exergonic. Maintenance of cell homeostasis depends on mechanisms that adjust the ATP generation processes according to the demand for energy. Thus, the function of cell bioenergetics systems requires that the ATP produced and sent to the sites of use – the ATPases – be finely coupled with the removal of products of its hydrolysis – adenosine diphosphate (ADP), Pi and H^+^ – so as to avoid the inhibition of ATPases, allowing continuity of biological processes.

Phosphocreatine (PCr) was discovered in 1927 in muscle tissue. Free creatinine (Cr) was generated from its breakdown during muscle contraction.^(1,2)^ In 1962, Cain and Davies inhibited the enzyme responsible for transforming PCr into Cr - creatine kinase (CK),and noted that the levels of ATP were rapidly reduced to the point that muscle contractions could no longer occur.^(3)^ Today it is clear that the PCr system is fundamental in promoting rapid resynthesis of ATP, by means of CK action (Equation 1).

(Equation 1)
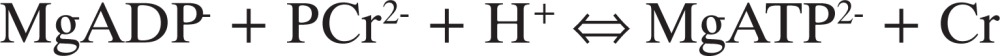


Since the PCr/CK system has a high level of ATP generation, it is particularly important in situations of high metabolic demand, such as high-intensity physical exercise, when the rate of ATP use exceeds its capacity for generation by other metabolic pathways.

### The phosphocreatine “shuttle” system

In 1970, Gudbjarnason et al. noted that in skeletal muscle submitted to ischemia, contractile activity was interrupted when PCr was depleted, despite the levels of ATP being reduced by only about 20%.^(4)^ The authors suggested that intracellular ATP may not be homogeneously distributed in muscle cells or be capable of rapid and efficient diffusion.

Four different isoforms of CK were located with distinct subcellular locations: cytosolic isoforms CK-M and CK-B (from muscle and brain, due to the tissue in which they were first identified) and two mitochondrial isoforms (sarcomeric CKmit, found in muscles, and ubiquitous CKmit, in the rest of the tissues). *In vivo*, the cytosolic isoforms combine into dimers, forming CK-BB, CK-MB, and CK-MM; the latter is predominant in skeletal muscle.^(5)^ These findings, added to the first experiments demonstrating that the supply with Cr stimulated mitochondrial respiration, showed that the mitochondrial and cytoplasmic compartments were interconnected by an organized energy transfer system constituted by different isoforms of CK. Thus, the foundations for the so-called PCr “shuttle” theory were established, formally proposed by Bessman in 1972.^(6)^

**Figure 1:**
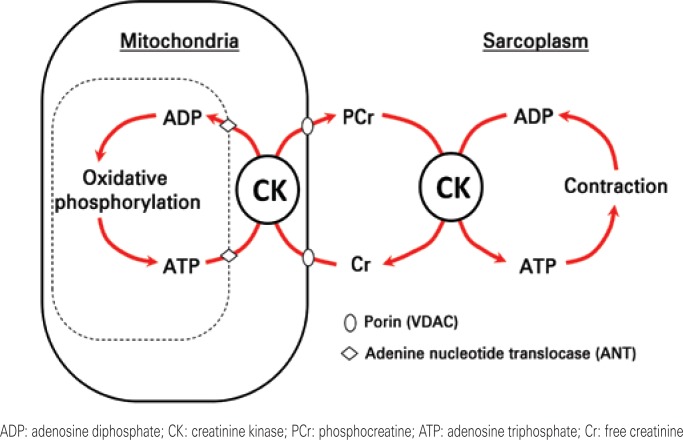
Phosphocreatine “shuttle” system

Adapted from: Neubauer S. Influence of left ventricular pressures and heart rate on myocardial high-energy phosphate metabolism. Basic Res Cardiol. 1998;93 Suppl 1:102-7. Review.

In the PCr “shuttle” system (Figure 1), high-energy phosphate is transferred from the ATP formed by means of oxidative phosphorylation in the mitochondria (production site) to Cr, via action of the CKmit, thus generating PCr and ADP. PCr diffuses into the cytoplasm, where under the action of cytosolic isoforms of CK, it generates ATP and Cr. ATP is then used by the ATPases (locations of use), while Cr returns to the interior of the mitochondria. This more easily crosses the mitochondrial membrane than the nucleotides of adenine, besides being present at higher levels in the intracellular medium. By means of this “shuttle” system, PCr performs another important function: it participates in the transfer of high-energy phosphate, present in ATP, from mitochondria to cytosol.^(7,8)^

The primary chemical reactions that use ATP in the skeletal and heart muscle are associated with the excitation-contraction coupling: myosin ATPase, in the microfibrils, and Ca^2+^-ATPase, in the sarcoplasmic reticulum (SR). Additionally, a major part of ATP is hydrolyzed by Na^+^/K^+^- ATPase in the sarcolemma. It has been demonstrated that CK is located in these sites, coupled, in a functional manner, to the ATPases.^(8)^ Therefore, it is clear that the location of the CL isoforms is fundamentally important for the system to work in an adequate manner, *i.e.*, for the ATP produced in the mitochondria to be effectively used by the cytosolic ATPases.

### Functional coupling system function for cell energy homeostasis

As was discussed, data point to the existence of limiting factors for the free diffusion of ATP in the cytoplasm. Today we know that the first barrier to molecule diffusion in the intracellular medium is macromolecular agglomeration, which refers to the high concentrations of macromolecules in the interior of cells. The concentration of proteins in the cytoplasm is about 200 to 300mg/mL, which corresponds to 20 to 30% of the intracellular volume.^(9,10)^ In the mitochondria, this density is even higher, with mitochondrial proteins occupying about 60% of the matrix volume.^(11)^ This high viscosity medium may cause a decrease in volume available for free diffusion of substrates,^(12)^ thus determining the activity of many cell processes.^(13)^ This is why the results of enzymatic studies that use solutions diluted with isolated enzymes (cell-free systems) may not correspond to the intact condition of the cell.

Another barrier to the mobility of metabolites in the cell microenvironment is the bonding of these molecules.^(14)^ Szent-Györgyi and Prior^(15)^ demonstrated that most of the ADP remains firmly connected to units of actin of the cytoskeleton of the cell, showing, once again, the importance of the phosphate transfer network, since cytosolic ADP cannot move to the mitochondria in which it exerts control over the mitochondrial respiration rate. This problem is overcome by the functional coupling system accomplished by the PCr “shuttle”.

Cr and PCr are smaller molecules, with a small negative charge or none at all when compared to adenine nucleotides. Experiments with magnetic nuclear resonance demonstrated that the mean distance of diffusion (indicator of the capacity of molecule diffusion) of PCr and Cr (57 and 37*μ*m) is much higher than that of ADP and ATP (1.8 and 22*μ*m, respectively).^(16)^ It is clear that among these metabolites, ADP is the limiting one as to the potential for diffusion through cytosol. Wallimann et al. reported that this potential, despite being low, is still sufficient to maintain the metabolic capacity in small cells or when the distance between the mitochondria and the cytosolic ATPases is not high, which does not match the properties of skeletal and cardiac muscles, making the energy transfer system through CK significant in these cells.^(8)^

While the agglomeration of macromolecules seems to be the primary mechanism of mobility restriction of larger solutes, bonding may be the most important mechanism in the case of smaller solutes.^(14)^ Since the production site of ATP in oxidative metabolism is in the interior of the mitochondria, and the locations of use are in the cytosol, the overlapping mechanisms of the barriers imposed by the low diffusion coefficients of adenine nucleotides and by the small availability of free ADP are essential. Obstacles coming from macromolecular bonding and agglomeration may be surpassed thanks to a mechanism of fine control and regulation of the metabolism, based on microcompartmentation and functional coupling.^(10)^

The spatial organization system, which allows interaction of enzymes, transporters, and their substrates in a supramolecular manner, is called functional coupling. This system is determined by two mechanisms that act in synergy in the accumulation and transfer of metabolites in small intercellular spaces, conferring greater efficiency to metabolic processes: metabolic canalization and cellular microcompartmentation.

By definition, compartment is a subcellular region in which biochemical reactions are kinetically isolated from each other.^(17)^ Inside the cells, the metabolism depends on the structural organization of the enzymes, forming microcompartments. Since the cells contain distinct microenvironments, the measurement of metabolite concentration in the whole cells or in certain tissues gives us an idea of the mean cell concentration, but not of the concentration of the metabolite under study which is effectively available at the active site of a particular enzyme. Therefore, cellular compartmentation may provide an additional control mechanism for metabolic pathways.

On the other hand, by means of metabolic canalization, an intermediate is transferred between adjacent enzymes, and depends less on cytosol diffusion. For example, it has been noted that the mean concentration of oxaloacetate in the mitochondrial matrix is much lower than necessary to support the function of the Krebs cycle. However, the high concentration of oxaloacetate, in the same microenvironment in which citrate synthase is located, allows the maintenance of speed of this cycle.^(13)^ This illustrates the importance of metabolic canalization and how cellular metabolism depends on the structural organization of the enzymes and intermediates, forming microcompartments.

In the PCr/CK system, the functional coupling performs so that the different isoforms of CK can act in two distinct directions. While CKmit acts in the direction of PCr synthesis, the cytosolic isoforms of CK act in the direction of local formation of ATP, around the ATPases. This was first demonstrated by mathematical calculations^(18)^ and then confirmed experimentally by means of magnetic resonance techniques with radioactive phosphorus.^(19)^

### Implications of changes in the phosphocreatine/creatinine kinase system

Some studies demonstrated that compromise of the PCr/CK system is related to a few functional alterations, especially in the myocardium. For example, transgenic mice that do not express guanidinoacetate-N-methyltransferase (GAMT), an enzyme necessary for synthesis of Cr, showed a reduced inotropic reserve and increased susceptibility of lesion by ischemia/reperfusion due to deficiency of Cr and PCr.^(20)^ Additionally, the myocardium of *knock-out* mice for the CKmit and CK-M isoforms is incapable of maintaining the levels of ATP when the animals are submitted to an intense exercise protocol, despite the baseline levels not showing modification. Also, these mice exhibit a significantly reduced free energy value (ΔG) for ATP hydrolysis during effort.^(21)^ This demonstrates that, in these animals, for which the total activity of CK is almost null, the increase in load imposed on the myocardium makes it more costly to generate work, reducing the free energy available with the hydrolysis of ATP. However, recent studies have raised questions as to the importance of the PCr/CK system in the myocardium at rest or during light to moderate intensity exercise. For example, Lygate et al.^(22)^ demonstrated that *knock-out* mice for GAMT showed no decline in voluntary locomotor activity and in capacity for exercise until exhaustion when compared to control animals, despite the deficiency of Cr. Furthermore, Branovets et al.,^(23)^ using cardiomyocytes of animals with GAMT deficiency, concluded that PCr is not essential for cardiac function at rest. It is possible that the deficiency in PCr may cause contractile damage only in supramaximal exercise, but additional research is necessary to elucidate this issue.

It was also demonstrated that pathological conditions that lead to cardiac hypertrophy, in man and in animal models, are characterized by a decrease in concentrations of Cr and PCr.^(24–29)^ In humans, a study demonstrated that the magnitude of the decrease of this content is directly related to the degree of insufficiency observed.^(26)^ Thus, compromise of the PCr system seems to precede the development of the contractile dysfunction, leading to the reduction of energy reserves available for regeneration of ATP and making the myocardium more likely to develop insufficiency.^(30)^

The mechanisms that result in the decrease of PCr concentration include lower expression of the Cr transporter^(27)^ and modifications in the pattern of expression of the CK isoforms, which lead to the reduction of the total levels of Cr and of the PCr/Cr ratio.^(21)^ Corroborating these findings, Ye et al.,^(31)^ using a model of congestive heart insufficiency in pigs, observed a decrease in the PCr/ATP ratio and in the flow through CK with a significant reduction in the expression of CK-M and CKmit. These and other studies demonstrate that the damage of the PCr/CK system seems to precede the development of the contractile dysfunction, leading to decreased energy reserve.^(32)^ The superexpression of the Cr transporter resulted in a moderate increase in the levels of Cr and glycogen, and protected the myocardium of the animals from acute infarction of the myocardium, with a 27% reduction in tissue necrosis, besides improved functional recovery after damage due to ischemia/reperfusion.^(33)^ Similarly, superexpression of the myofibril isoform of CK (CK-M) resulted in increase in ATP flow by CK and in improved contractile function in a model of cardiac insufficiency.^(34)^

Animals supplemented with β guanidyl-propionic acid (β-GPA, a competitor for Cr transport), which, however, show a significant depletion of Cr in the myocardium, also exhibit damage in cardiac function, especially with high workloads. In animals treated with β-GPA, increased concentration of free ADP makes ΔG for ATP hydrolysis less negative, rendering this reaction less efficient in terms of energy.^(32)^ Since the sequester of calcium depends on a highly negative ΔG, and that CK is in the sarcoplasmic reticulum functionally coupled with SR, the drop in levels of Cr in the myocardium seems to explain part of the cardiac alterations observed in individuals and animals that present with decreased reserve of Cr under pathological or experimental conditions.

This is the case of hyperthyroidism, in which there is an accentuated drop in the supplies of Cr.^(35)^It is known that, under these conditions, the heart shows a limitation in maximal work capacity, with an accentuated reduction in the levels of ATP as the energy demand increases.^(36,37)^ Maybe the decrease in offer of Cr to the myocardium is responsible for this limitation. It was demonstrated that at low and high rates of work imposed on the heart (measured by systolic peak pressure *versus* heart rate), the myocardium of hyperthyroid animals shows decreased levels of ATP and elevated levels of free ADP^(35)^, which suggests that the excess of thyroid hormones has affected the heart's working capacity, in part due to the decreased supply of Cr in the myocardium, parallel to the accentuated drop in expression of the Cr transporter. A study with hypophysectomized rats demonstrated that the thyroid hormone promotes an increase in the levels of PCr and in the PCr/Cr ratio in skeletal muscle, besides accelerating PCr regeneration after muscle contractions.^(38)^

On the other hand, in induction of the hypothyroid condition in rats for 3 weeks, Athéa et al.^(39)^ verified that it did not affect total CK activity, as well as the gene expression and activity of CKmit. However, the mitochondria of these animals showed a decrease in sensitivity to ADP and to Cr, evidenced by the constant increase of the Michaelis-Menten constant (Km_ADP_ of 189±12*μ*M to 228±12*μ*M and Km_ADP+Cr_ of 65±5*μ*M to 101±8*μ*M). In physiological terms, this means that more ADP and Cr are necessary under this experimental condition for the stimulation of the mitochondrial function. This effect may have occurred by the decrease of content of cardiolipin in the mitochondrial membrane, in which synthesis is stimulated by thyroid hormones.^(40)^ This phospholipids is important for the bond between CKmit and the mitochondrial membrane.^(41)^ In this way, it is presumed that under conditions of hypothyroidism, the PCr “shuttle” system acts at a slower rhythm than under euthyroid conditions,^(22)^ which results in the lower availability of the ATP generated in the mitochondria for the locations of use in the cytoplasm. Khushu et al.^(42)^ demonstrated that despite the fact that patients with hypothyroidism display intramuscular levels of PCr similar to those of euthyroid individuals, the resynthesis of PCr, after exercise, is reduced, indicating a reduction in oxidative capacity in the skeletal muscle.

A recent study in our group demonstrated that supplementation with Cr promotes the reduction of reactive oxygen species (ROS) in skeletal muscle.^(43)^ The increase in ROS production occurs in various metabolic, neurologic, and endocrine diseases, among others, and treatment with Cr has proved to be an efficient strategy in a large part of these diseases.^(44)^ Additional research should determine the true therapeutic potential of Cr supplementation.

## CONCLUSION

The understanding of the functional coupling phenomenon, involving cellular microcompartmentation and metabolic canalization, may help to better comprehend the integration among the various cellular systems of adenosine triphosphate generation that interact to maintain skeletal and cardiac cell homeostasis. In this regard we show that the regulation of cellular energy metabolism is intimately related to the organizational structure of the cell components. Such evidence brings to light part of the function, many times ignored, of the phosphocreatine/creatinine kinase system, which contributes towards a better understanding of muscle physiology as well as relevant clinical aspects. Additionally, they point to relevant issues that still deserve more investigation in the field of cellular bioenergy.
